# Perspectives on family-based suicide prevention and postvention

**DOI:** 10.1192/bjo.2022.532

**Published:** 2022-07-07

**Authors:** Karolina Krysinska, Karl Andriessen

**Affiliations:** Centre for Mental Health, Melbourne School of Population and Global Health, The University of Melbourne, Melbourne, Australia; Centre for Mental Health, Melbourne School of Population and Global Health, The University of Melbourne, Melbourne, Australia

**Keywords:** Suicide, family interventions, intervention, effectiveness, suicide prevention

## Abstract

Suicide remains an important public health problem worldwide. Many countries have developed national suicide prevention policies or guidelines, which often include family-based recommendations regarding suicide prevention, intervention or postvention. A recent systematic review, published in this journal, failed to find evidence of an impact of family-based recommendations in national guidelines on national suicide rates. In this editorial, we review other studies providing promising evidence of effectiveness of family-based interventions in the field of suicide prevention and postvention, and note that further studies are needed, especially in adult and older adult populations.

Each year more than 700 000 people die by suicide, making it a major public health problem worldwide.^1^ Research has identified various risk and protective factors, including increased vulnerability within families, cognitive characteristics, mental health problems and socioeconomic variables.^1^ Suicide is an act of an individual; however, it rarely occurs within an interpersonal vacuum. Often, family members are informal carers of a suicidal person. Thus, comprehensive suicide prevention policies and guidelines should ensure that adequate resources and support are available to the informal carers.

Many countries have developed national suicide prevention policies or guidelines. Recently, Panesar and colleagues^2^ conducted a systematic review of 62 guidelines from 46 countries that included at least one family-based recommendation regarding suicide prevention, intervention or postvention. The study did not find a statistical difference in suicide rates between countries that had included any family-based recommendations, nor between countries that had included one, two or all three categories of family-based recommendations. Still, the authors reported a beneficial tendency in suicide rates in countries that had included all three categories, inspiring them to conclude that national suicide prevention policies must include all categories of family-oriented approaches.^2^

Although the conclusion of the study has face validity and is in line with World Health Organization (WHO) recommendations for country-based suicide prevention policies,^1^ the study entailed important limitations.^2^ The number of family-based recommendations in the reviewed national guidelines ranged between one and eleven. There was also a broad range of recommendations within each category (prevention, intervention, postvention), indicating that the type of family-based recommendations differed across countries (e.g. education, psychotherapy). Moreover, the notion of ‘family’ may also differ across countries. The study involved guidelines published since the year 2000, and the most recent suicide rates of countries as available from the WHO in 2019. However, the cross-sectional study did not account for the time since publication of the guidelines. Further, the study assumed a causal relationship between the inclusion of family-oriented suicide prevention recommendations and national suicide rates, which is hard to assess in the absence of information about other components of the suicide prevention guidelines or how guidelines have been implemented.^3^

Thus, the study seems to have been based on the expectation that the presence of family-based suicide prevention recommendations in national guidelines would positively affect suicide mortality rates. Given that the hypothesis was not confirmed, it would be easy to dismiss family-oriented suicide prevention. However, the study design may have hindered capturing potential evidence, available from various studies in suicide prevention and postvention, such as studies that have focused on other measures of effectiveness of interventions.

## Prevention

Several promising psychosocial interventions involving the family have been developed over the past three decades for people (mostly adolescents) who have survived a suicide attempt or reported suicidal ideation.^4–7^ Still, there is no one standard ‘family-based’ prevention programme or psychosocial intervention, with established effectiveness.^4^ Diamond and colleagues^5^ reported great variation in ‘how and to what degree’ the established treatments for young people at risk for suicide include a family component. This can range from individual treatments encompassing a family component to treatments that focus specifically on the family. There are differences regarding the specific family processes targeted (e.g. problem-solving, empathic parenting, self-regulation), the role of the family in treatment (e.g. facilitation of treatment versus a mechanism of change), the type of treatment modality (e.g. cognitive–behavioural therapy, family-based crisis intervention, attachment-based family therapy), treatment duration (e.g. a single session versus over 12 months) and intervention outcomes measured (e.g. suicidal ideation, suicide attempt, depression, family functioning).^6^ Further, family-based interventions can be conducted in various settings, such as a hospital, out-patient services following a hospital stay, or out-patient treatment.^4^ This raises a question whether a relatively crude measure, such as national suicide rates,^2^ can adequately reflect effectiveness of the various existing family-based prevention and intervention programmes, even assuming that the national suicide management guidelines have been properly implemented. Furthermore, looking at national suicide rates does not allow to measure the impact family-level variables may have had on suicidal ideation or suicide attempts.

Family-based interventions tend to target young people at risk of suicide, and there is a significant lack of family-based treatments for adults, including older adults. In a recent scoping review, Sullivan and colleagues,^6^ found only one controlled trial of a family treatment for individuals at risk of suicide that included adults. The other nine trials targeted exclusively young people under 18 years of age. In a review by Frey and Hunt,^4^ none of the 16 studies that tested 13 family-based interventions included adults with suicidal ideation or behaviour. Consequently, there is a question whether an analysis of youth suicide rates in countries that have included family-based recommendations in their guidelines, instead of suicide rates for the total population,^2^ might have yielded different results.

The picture is further complicated by methodological limitations of effectiveness studies of family-based treatments for suicidal thoughts and behaviour. Brent and colleagues^7^ drafted specific recommendations for future intervention studies in this field, which include ensuring sufficient study power; use of consistent and accepted definitions of suicidality; ensuring correct timing, sufficient intensity and duration of treatment; and targeting specific clinical outcomes (e.g. family processes, attachment, sleep, positive affect). Further, the evidence base for the effectiveness of family-based intervention in non-Western countries and non-urban locations is practically non-existent.^6^ One can hope that methodologically strong intervention studies will inform and guide implementation of national suicide management guidelines, which will make a significant difference in regard to the much-coveted outcome of lower suicide mortality.

Another possible challenge in the implementation of family-based suicide prevention interventions and treatments is the complexity of family environments of people at risk. Family members of people at risk of suicide, including those reporting chronic suicidal ideation and multiple suicide attempts, may not have the motivation or may not be prepared to engage in family-based treatment.^4^ Family carers often need support themselves, and there are only limited data on existing interventions for informal carers and their effectiveness.^8^ Adults at risk for suicide can be socially isolated and thus not able to identify a support person, such as a family member, who can be included in treatment.^4^ As such, Frey and Hunt^4^ recommend that ‘family based interventions must include ways to access and treat these patients [at risk of suicide] before complete isolation or family burnout occurs’.

## Postvention

Traditionally, postvention has been focused on individuals bereaved by suicide, Nonetheless, the literature indicates that individual grief affects family functioning and *vice versa*.^9^ Recent research with adolescents bereaved by suicide revealed how the bereavement can rupture the family equilibrium, and how adolescents and parents commonly experienced worries and struggles regarding how to support each other.^10^ Still, they also engaged with each other to find a new family balance. These findings are in line with review of the literature and consensus recommendations, indicating that a parental or family component may contribute to effectiveness of support offered to children and adolescents bereaved by suicide.^11^

Overall, studies have reported mixed findings regarding the effectiveness of postvention interventions, and especially the effectiveness of interventions in regard to risk for suicidal ideation and behaviour remains unclear, mostly because of a lack of research.^12^ Nonetheless, a few family-oriented studies reported on suicidal ideation as an intervention outcome. Wittouck and colleagues^13^ found no differences between study groups regarding suicidal ideation immediately after completion of therapy as well as 8 months postintervention. De Groot and colleagues^14^ found no differences between family-based psychotherapy and treatment as usual on measures of suicidal ideation.

Sandler and colleagues^15^ reported long-term results of a family bereavement programme offered to children and adolescents who had lost a parent by illness, accident, homicide or suicide. The programme consisted of 12 group sessions for caregivers and children/adolescents, as well as two individual sessions. Caregiver sessions focused, for example, on parenting skills, whereas youth sessions focused on effective coping skills. Participants reported less suicide ideation and/or attempts at the 6- and 15-year follow-up evaluation compared with a literature control condition.

## Implications

Despite the progress that has been made over the recent decades regarding family-based suicide prevention and postvention interventions, several questions concerning their effectiveness remain. Further studies are needed, especially regarding suicide-related outcomes in adults and older adults. There is also a clear need to document the type of interventions, the notion of ‘family’ adopted in the studies and how interventions have been implemented and/or embedded in broader national suicide prevention guidelines or policies. Such basic data across countries are needed to further examine the potential impact of family-based interventions on suicide mortality and risk and protective factors for suicidal behaviour, including coping with psychosocial problems.
